# Heritable breast cancer in twins

**DOI:** 10.1038/sj.bjc.6600429

**Published:** 2002-08-01

**Authors:** T M Mack, A S Hamilton, M F Press, A Diep, E B Rappaport

**Affiliations:** Department of Preventive Medicine, University of Southern California Keck School of Medicine, Norris Comprehensive Cancer Center, 1441 Eastlake Avenue MC9175, Los Angeles, California, CA 90089-9175, USA; Department of Pathology, University of Southern California Keck School of Medicine, Norris Comprehensive Cancer Center, 1441 Eastlake Avenue MC9175, Los Angeles, California, CA 90089-9175, USA

**Keywords:** breast neoplasms, genetics, etiology, pathogenesis, twins

## Abstract

Known major mutations such as BRCA1/2 and TP53 only cause a small proportion of heritable breast cancers. Co-dominant genes of lower penetrance that regulate hormones have been thought responsible for most others. Incident breast cancer cases in the identical (monozygotic) twins of representative cases reflect the entire range of pertinent alleles, whether acting singly or in combination. Having reported the rate in twins and other relatives of cases to be high and nearly constant over age, we now examine the descriptive and histological characteristics of the concordant and discordant breast cancers occurring in 2310 affected pairs of monozygotic and fraternal (dizygotic) twins in relation to conventional expectations and hypotheses. Like other first-degree relatives, dizygotic co-twins of breast cancer cases are at higher than usual risk (standardised incidence ratio (SIR)=1.7, CI=1.1–2.6), but the additional cases among monozygotic co-twins of cases are much more numerous, both before and after menopause (SIR=4.4, CI=3.6–5.6), than the 100% genetic identity would predict. Monozygotic co-twin diagnoses following early proband cancers also occur more rapidly than expected (within 5 years, SIR=20.0, CI=7.5–53.3). Cases in concordant pairs represent heritable disease and are significantly more likely to be oestrogen receptor-positive than those of comparable age from discordant pairs. The increase in risk to the monozygotic co-twins of cases cannot be attributed to the common environment, to factors that cumulate with age, or to any aggregate of single autosomal dominant mutations. The genotype more plausibly consists of multiple co-existing susceptibility alleles acting through heightened susceptibility to hormones and/or defective tumour suppression. The resultant class of disease accounts for a larger proportion of all breast cancers than previously thought, with a rather high overall penetrance. Some of the biological characteristics differ from those of breast cancer generally.

*British Journal of Cancer* (2002) **87**, 294–300. doi:10.1038/sj.bjc.6600429 www.bjcancer.com

© 2002 Cancer Research UK

## 

The prevalence of familial cases indicates that about 10% of all breast cancers are heritable (Rowell *et al*, 1994), but major mutations such as BRCA1, BRCA2, and TP53 can only be a minority of these (Cui *et al*, 2001b; Peto *et al*, 1999); familial cases are especially prominent before menopause (Pharoah *et al*, 1997). The genes responsible for most heritable breast cancers are unidentified. Due to the role of hormones in breast cancer risk, genes regulating hormone production or transport have been emphasized. Those that are common and of low penetrance have been singled out (Ford *et al*, 1995; Feigelson *et al*, 1996), and an autosomal mode of inheritance is thought likely (Williams and Anderson, 1984; Newman *et al*, 1988; Iselius *et al*, 1991; Cui *et al*, 2001a; Claus *et al*, 1991; Chen *et al*, 1995; Bishop *et al*, 1988).

Although BRCA1 tumours are less likely to express oestrogen receptors (Armes *et al*, 1999; Phillips, 2000), heritable tumours seem histologically heterogeneous (Lakhani *et al*, 2000). Attempts to separate non-heritable from heritable breast cancer cases have relied on family history, and even when based on first-degree relatives, this criterion is unsatisfactory. Many genetically determined cases give a false negative family history (Cui and Hopper, 2000), and false positive histories occur by chance, especially in large families. Even BRCA1/2 mutations do not correlate particularly well with family history (Hopper *et al*, 1999). Moreover, true multiplex families mostly reflect conditions of high penetrance. Cases caused by gene combinations, recessive genes, or genes of low penetrance are less likely to have affected relatives. Thus neither the true proportion of breast cancers that are heritable nor the proportionate role of specific genetic determinants is actually known.

Adult twins are ordinary persons who share a nearly identical early environment as well as about half (dizygotic) or all (monozygotic) of the genome. Monozygotic (MZ) twinning is not known to be appreciably influenced by either genetic or environmental factors. Therefore the cases of disease among MZ twins can be presumed genetically representative of the population, and disease-concordant pairs must necessarily share all heritable determinants. We have reported (Peto and Mack, 2000) that the subsequent annual incidence in the MZ co-twins of breast cancer cases is not only extremely high (1300 : 100 000) but relatively constant throughout life, just as it is in the contra-lateral breasts of cases (Robbins and Berg, 1964; Hislop *et al*, 1984; Harvey and Brinton, 1985). Here we assess in more detail the breast cancers occurring in the MZ and DZ twins of representative breast and other cancer cases (Mack *et al*, 2000), report the unique descriptive and histological characteristics of concordant twin breast cancer cases, and examine current hypotheses in light of the findings.

## METHODS

From 1980–91, 17 245 affected twin pairs responded to advertisements seeking ‘twins with cancer and other chronic diseases’ in the non-classified pages of major periodicals serving English-speaking North America (Mack *et al*, 2000). Each pair was contacted by telephone and asked to provide details of their age, zygosity, and diagnosis.

Among the 6325 female twin pairs with cancer were 2562 probands with breast cancer, including the members of 200 MZ and 109 dizygotic (DZ) concordant pairs. More than 95% were non-Latino and white, and we have assessed their representiveness by computing the estimated prevalence of breast cancer cases among living white adult twins in North America (Mack *et al*, 2000), using cohort-specific birth rates (Elwood, 1973; Jeanneret and Macmahon, 1962; Statistics NCfH, all years), life tables (Kleinman *et al*, 1991), and site-specific cancer incidence (Gaudette, 1992; Hankey and Percy, 1992) and survival (Hankey *et al*, 1993). We also compared the unaffected co-twins to population-based samples of healthy US residents (Mack *et al*, 2000). We estimate that we identified over a third of all MZ twin breast cancer cases occurring before age 60 in the period. Our ascertainment was less complete for twin cases who were DZ, over 65 at diagnosis, or discordant for disease at the time of ascertainment, but neither region, community characteristics, interval since diagnosis, nor outcome appeared to influence ascertainment (Mack *et al*, 2000).

Medical records were sought to verify diagnoses, and were obtained for 80% of the breast cancer cases. Of those, 94% were reported to be invasive. Tumour specimens were requested for review (successfully in 65%) and cases were classified according to standard cancer registry practice (Percy *et al*, 1990). When diagnostic and classificational errors were found to be negligible among the first 805 specimens reviewed, the practice was discontinued. Twins' perception of their zygosity, repeatedly shown to be over 90% accurate by others (Kasriel and Eaves, 1976; Torgersen, 1979) as well as ourselves (Deapen *et al*, 1992; Kumar *et al*, 1993), were nearly all in agreement, and those in disagreement were excluded from zygosity-specific results.

All twins were followed prospectively by mail to identify deaths and new diagnoses. National age, period, and neoplasm-specific incidence rates (Hankey and Percy, 1992), were applied to the person-years of follow-up to estimate the expected number of new cases. The indirectly age-adjusted standardised incidence ratio of observed to expected cases (SIR), was calculated by age, sex, and zygosity, as was the incidence rate/100 000 person-years. Since inclusion of some co-twins preferentially identified as cases in retrospect at original ascertainment may introduce bias, pairs already concordant at ascertainment were excluded. Thus analysis was restricted to the initially unaffected 2310 co-twins of breast cancer cases and 3628 co-twins of other cancer cases. Events occurring between ascertainment of the affected pair and the date of last contact, always prior to February 1, 1993, were recorded. For MZ twins of breast cancer cases, the average length of follow-up was 4.8 (95% CL 4.6–5.0) years, with 16.4 and 49.0% followed for one year or less and 5 or more years respectively. For DZ twins of breast cancer cases, the average follow-up was for 4.6 (95% CL 4.4–4.8) years, with 14.9 and 48.8% followed for 1 or less and 5 or more years respectively.

For concordant pairs (including those concordant at ascertainment) and a sample of MZ discordant pairs, additional efforts were made to obtain representative tumour blocks from the initial breast cancer surgery. Using the paraffin-embedded tissue from 196 cases from concordant pairs and 190 cases from MZ discordant pairs, a single pathologist (MP) blindly reviewed the histology of the tumours, and assessed the prevalence of oestrogen receptors (ER), progestin receptors (PR), p53, and HER-2/neu expression by immunohistochemical methods. HER2/neu membrane protein was scored as low, over-expressed, or highly over-expressed, and considered positive under either of the latter alternatives. ER, PR, and p53 nuclear proteins were scored by prevalence of cells staining at each of three levels of intensity. For the present purpose a tumour was considered to be positive if 10% or more of the cells stained positively. Multivariate linear regression analysis (SAS Proc GLM) was used to control for age at diagnosis when comparing the frequency of positive tumour markers among the twin pair subsets.

Religious preference was obtained by questionnaire from the twins comprising 1944 affected pairs. We located and obtained blood samples from 27 surviving cases belonging to 19 multiplex Jewish families in which diagnoses occurred before 50. Three common Ashkanazi mutations: two BRCA1 (185delAG, exon 2, and 5382insC, exon 20) and one BRCA2 (6174delT, exon 11) were tested (Ursin *et al*, 1997).

## RESULTS

One hundred and forty-eight cancers of the breast in the initially healthy co-twins of cancer cases were diagnosed during the period of prospective follow-up, of whom 99 (22 DZ and 77 MZ) occurred among the co-twins of breast cancer cases. Table 1

**Table 1:**
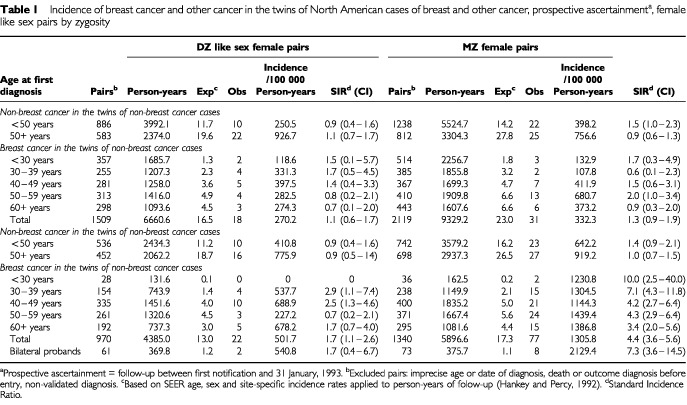
Incidence of breast cancer and other cancer in the twins of North American cases of breast and other cancer, prospective ascertainment^a^, female like sex pairs by zygosity

describes the occurrence of these prospectively identified cancers in terms of incidence and standardised incidence ratio according to zygosity, age and site of proband diagnosis, and person-years of follow-up.

**Figure 1:**
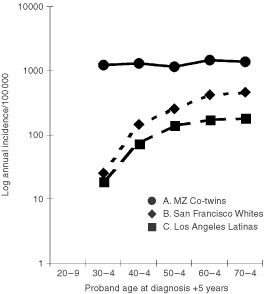
Age-specific Incidence of breast cancer in identical co-twins of breast cancer cases compared to that in highest and lowest risk North American populations (42).

Breast cancer incidence in the co-twins of non-breast cancer cases increased with age past menopause as expected, and for all ages combined, no substantial or significant risk attributable to twin status was found. Marginal excesses of malignancy other than breast cancer occurred in MZ, but not DZ, twins of breast cancer cases diagnosed before age 50. The appearance of additional breast cancer cases among the initially healthy co-twins of breast cancer cases was substantial and significant. Among the DZ co-twins, the 22 new cases represent an unstable annual age-specific incidence ranging from 227 to 689 : 100 000, reflecting a statistically significant age-adjusted SIR of 1.7, a 70% excess over expected. Among the MZ co-twins, the 77 new cases reflect an annual incidence ranging from 1144 to 1439 : 100 000, and an overall SIR of 4.4, a 340% excess over expected, and one as high as 7.1 before 40 years of age. Figure 1

compares the age-specific rates in the MZ co-twins to the highest and lowest North American population-based rates (Parkin *et al*, 1992). If the MZ proband had bilateral breast cancer, especially likely to represent heritable disease (Bernstein *et al*, 1992; Hislop *et al*, 1984), the SIR for the co-twins showed a similar gradient with age and was even higher overall at 7.3, reflecting an attributable excess of 630%. Overall, 23% and 3% of the concordantly affected MZ pairs had one and more than one affected first degree relative respectively (data not shown).

In Table 2

**Table 2:**
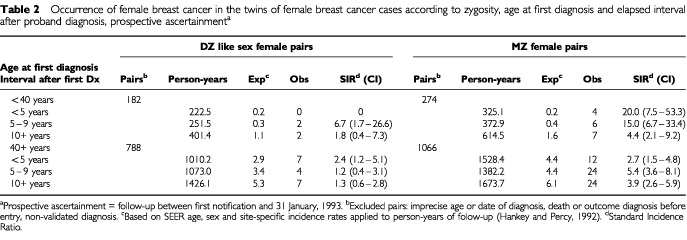
Occurrence of female breast cancer in the twins of female breast cancer cases according to zygosity, age at first diagnosis and elapsed interval after proband diagnosis, prospective ascertainment^a^

, the appearance of breast cancer in co-twins is described according to the interval following the proband's cancer diagnosis. Within the first five years after the diagnosis in a proband younger than age 40, the SIR among MZ co-twins was many times higher than expected, and the magnitude fell in inverse proportion to the time elapsed. This tendency was not apparent among DZ co-twins or after diagnoses in older probands.

Overall, 8.6% of the concordant and 7.6% of the discordant MZ pairs identified themselves as Jewish. Among those first diagnosed before age 50, 10.1% of those concordant and 6.8% of those discordant did so. Thus being Jewish increased the risk of concordant diagnoses by a factor of 1.3 overall, and 1.5 premenopausally. Of the 19 tested multiplex Jewish families with at least one premenopausal diagnosis, BRCA1 mutations were found in 4, and a BRCA2 mutation in 1.

More than 97% of the tissue samples from MZ twins (concordant and discordant) showed evidence of invasiveness (Table 3

**Table 3:**
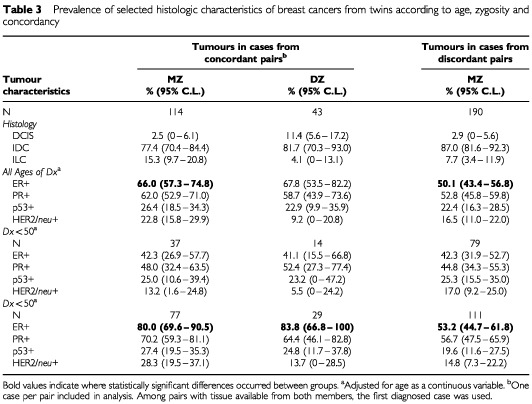
Prevalence of selected histologic characteristics of breast cancers from twins according to age, zygosity and concordancy

) Although a slightly higher proportion of lobular tumours occurred among the MZ concordant pairs, no significant difference by histologic subtype (ductal, lobular, ductal *in-situ*) was found, and only a single tumour (in a DZ twin) showed a medullary histopathology. PR and ER positivity were significantly more common among older cases, and ER positivity was significantly higher among cases from concordant than among those from discordant pairs, especially after age 50 (Figure 2

**Figure 2:**
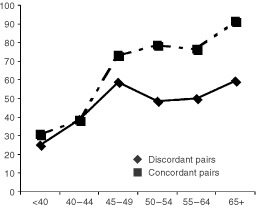
Percentage of tumours ER+ by age at diagnosis: MZ cases from breast cancer concordant and discordant pairs.

). After age 50, HER2/neu and p53 were more common among cases from concordant than discordant MZ pairs; the former difference was significant and the latter nearly so. Biomarker combinations were examined, and concordant cases were more likely to be ER+PR+ (age-adjusted prevalence=54.1%1.7% MZ, 55.07.2% DZ) than were discordant cases (41.30.8% MZ), but the difference could be explained by chance. No excess prevalence of ER-p53+ concordant cases was found (concordant MZ 13.21%, DZ 12.84.6%, and discordant MZ 13.914.4%).

## DISCUSSION

Since DZ co-twins experience a level of risk no higher than that of other first-degree relatives generally (Brinton *et al*, 1982; Claus *et al*, 1990; Houlston *et al*, 1992; Olsen *et al*, 1999; Thompson, 1994; Tulinius *et al*, 1992), despite sharing a substantially more similar environment, the very high incidence among representative MZ co-twins of breast cancer cases (Table 1) serves to verify the substantial heritability of this disease (Easton *et al*, 1993; Eby *et al*, 1994; Lichtenstein *et al*, 2000; Thompson, 1994). Moreover, our estimate of incidence in MZ co-twins is probably an underestimate. It is based solely on prospective follow-up, and a number of rapidly concordant MZ pairs, i.e. those with co-twin cases occurring soon after the proband diagnosis but before ascertainment, were preferentially excluded to eliminate bias. The similarity of the observed high and constant age-specific rate to that in the contralateral breasts of breast cancer cases (Harvey and Brinton, 1985; Robbins and Berg, 1964) provides additional evidence that bilateral disease is largely attributable to the genome rather than the personal environment.

No more than a small minority of heritable co-twin cases can be attributed to BRCA1/BRCA2 or other known major mutations. More than three-fourths of the additional cases among MZ co-twins occur after 40 (Table 1), whereas most BRCA1/2 cases occur before that age (Ford *et al*, 1998). While 23% of the concordantly affected MZ pairs had one affected first degree relative, only 3% had more than one, in strong contrast to BRCA1/2 cases (Cui and Hopper, 2000). Jewish women are at high risk from disease caused by major mutations. Although the risk to a woman from a Jewish multiplex family (Egan *et al*, 1996) or a Jewish family with a BRCA1/2 mutation (Fodor *et al*, 1998) is increased by a factor of 3–4, and although Jewish women are also at higher risk from additional breast cancer risk factors (Swift *et al*, 1987), we found that a Jewish MZ co-twin's risk of becoming affected was increased only marginally more than that to Jewish women generally (Mack *et al*, 1985; Warner *et al*, 1999). Even among multiplex Jewish families with early cases, we could identify only a few with BRCA1/2 mutations. Moreover, whereas BRCA1/2 neoplasms tend to be ER-, especially in connection with P53 mutations, and tend to include an excess of the medullary histological type (Lakhani, 1999; Phillips, 2000), the tumours in these concordant MZ twins were not medullary, and tended to be ER+, without a link to P53 mutations (Table 3, Figure 2).

The proportion of MZ co-twin cases attributable to genetic determinants is roughly 77% (4.4-1)/4.4) (Table 1), indicating that MZ twin breast cancer-concordant cases, unlike familial cases generally, are much more likely than not to represent heritable cases. Based on the observed age-specific incidence in the co-twins of MZ cases (Table 1), the cumulative risk among those surviving to age 75 would be at least 44.5%, indicating that only a fraction of the minority with heritable disease remain discordant. MZ twin breast cancer-discordant cases can therefore be presumed to represent disease which is not strongly heritable. Material from breast cancer concordant and discordant MZ twin pairs clearly offers the best opportunity available to compare cases that are heritable with those that are not.

Our results suggest that heritable breast cancer represents a larger proportion of the total burden than conventionally thought. In Scandinavia (Lichtenstein *et al*, 2000), 14% of MZ twin pairs were found to be concordant. However, the population-based ascertainment ignored discordant mortality and necessarily excluded substantial numbers of subjects at the extremes of age at risk, as has been pointed out (Risch, 2001), indicating that the actual cumulative concordance exceeds 20%. If so, and if more than 77% of the cases in MZ twins represent heritable forms of disease, the proportion of all breast cancer represented by heritable disease exceeds 15%, and we have speculated on other grounds that it may be even higher (Peto and Mack, 2000).

The pattern of occurrence in MZ and DZ co-twins is determined by the mode of inheritance of disease. Any excess risk to a DZ twin of a case is the result of sharing 50% of the genome and a common early environment. Given a predominantly autosomal dominant mode of inheritance, the increment of risk to an MZ co-twin would be slightly less than double that to a DZ co-twin. This is because the additional risk from sharing the other half of the genome would produce the same incremental contribution as that from sharing the first half, and little, if any, added environmental risk would be expected, because the commonality of MZ twins' early environment is probably only marginally greater than that of DZ twins. Since the DZ twin of a case suffers a 70% increase in risk (overall relative risk of 1.7 from Table 1), an increase of under 140% would be expected. In fact, a relative risk of 4.4 (Table 1) indicates an increase of at least 340%, and the confidence limits are such that the difference cannot be explained by chance. A similarly large increase in risk attributable to MZ status can be calculated from the Scandinavian twins study (Lichtenstein *et al*, 2000). Such a large increment indicates that most heritable breast cancers do not result from single autosomal dominant alleles. While a recessive mode of inheritance cannot be ruled out, that would seem an unlikely alternative, especially as a cause of older cases (Cui *et al*, 2001b). It is more likely that a substantial proportion of heritable breast cancer in both younger and older women is polygenic, resulting from the interaction of two or more coexisting alleles (Chen *et al*, 1995). In such a circumstance, a gene acting through a hormonal mechanism might only do so in the presence of another existing genetic error in, for example, a repair (tumour suppression) gene. Such a combination would explain how a general defect of molecular repair might only produce malignancy in one specific organ (Haber, 2000).

The pattern of occurrence in MZ twins also necessarily reflects the mechanism of heritable breast carcinogenesis. The shorter than expected intervals between co-twin diagnoses (Table 2) are similar to the chronologic sequence of primary and contra-lateral breast cancer diagnoses (Harvey and Brinton, 1985; Prior and Waterhouse, 1978; Robbins and Berg, 1964; Storm and Jensen, 1986) and, given the long latency, points to a roughly similar early age at the time of a crucial causal event.

Moreover, to attain an incidence level over 1200 : 100 000 before age 30 (Table 1), the age-specific incidence of strictly heritable disease must have risen very early and rapidly and must account for nearly every early case. While the rate in MZ co-twins, based on sporadic as well as heritable disease, stays virtually constant (Figure 1), the population-based rate, largely from sporadic cases, increases with age by an order of magnitude. The rate of heritable disease therefore could not increase much over the same period and may actually decline, another observation inconsistent with causation by age-specific hormone accumulation. Thus alleles such as those at CYP 17 (Henderson and Feigelson, 2000) are unlikely to be the predominant determinants of heritable disease. Nor is the polymorphism responsible for enzymatic conversion of androgens into oestrogens (CYP19) likely to play a major role in heritable disease, since this conversion takes place in fat cells, largely after menopause, and too late to explain the early excess in risk.

Thus genetically determined high hormone levels are probably not the predominant mechanism of heritable breast cancer carcinogenesis. In fact the focus on high hormone levels as the phenotypic expression of causal genes may be misplaced, because the familial aggregation of hormone levels may not be even principally genetic in origin. Lifestyle, including physical exercise (Bernstein *et al*, 1994), perinatal conditions (Ekbom *et al*, 1997, 2000), transient episodes of disease-induced catabolism, and deficiencies in early diet (Berkey *et al*, 2000; Willett, 1997), produce variations in age at maturation, and probably underlie most of the change in risk seen after migration (Shimizu *et al*, 1991) and economic development (Miller and Bulbrook, 1986). Because families vary in the ease and rapidity of acculturation and adaptation, age-specific hormone profiles are likely to vary between them.

An alternative mechanism of heritable susceptibility, a genetically determined high cellular sensitivity to reproductive hormones, is suggested by an animal model and does fit the observed pattern of occurrence. When treated with estradiol at a standard dosage, certain rat strains rapidly develop breast cancer almost without exception (Shull *et al*, 1997; Harvell *et al*, 2000). Cancers resultant from a genetically induced enhancement of human sensitivity to hormone exposure would be induced by the first major endogenous hormone exposure at puberty, resulting in a pattern of risk like that observed among MZ twins (Table 1). Such sensitivity might result from polymorphic variation in the human estrogen receptor (ER) gene (Andersen *et al*, 1994), or by either overexpression of a transcription co-activator or underexpression of a co-repressor (Ansick *et al*, 1997). Age-specific breast sensitivity is precedented by radiogenic carcinogenesis which is closely tied to early age at exposure (Aisenberg *et al*, 1997), and adolescent soy intake during adolescence may preferentially reduce breast cancer risk (Shu *et al*, 2001).

Twins comprise 2% of the North American population (Mack *et al*, 2000) and suffer over 20 000 cancer diagnoses each year. We found no unusual cancer risk to twins as twins, since no significant or substantial increase in overall relative risk of cancer to the twin of a case of another form of cancer appeared (Table 1). While there seems to be no difference between breast cancer risk to the DZ twin of a case and that to a non-twin sibling, the high risk to the MZ co-twin of an early breast cancer, being twice that of a second primary diagnosis, is of serious clinical concern. More frequent screening procedures, chemoprophylaxis, and possibly prophylactic surgery, with all corresponding pros and cons, should be discussed with each such woman, as if she were a high risk gene carrier. Unfortunately, twins rarely volunteer their twin status to their doctors. The question of twinship should be posed to every patient when the diagnosis of a serious familial disease is under consideration. Such a practice will become even more clinically important as our knowledge of heritable risk and gene/environment interaction expands.
